# Gating of Social Behavior by Inhibitory Inputs from Hippocampal CA1 to Retrosplenial Agranular Cortex

**DOI:** 10.1007/s12264-023-01172-0

**Published:** 2024-01-28

**Authors:** Yuhan Shi, Jingjing Yan, Xiaohong Xu, Zilong Qiu

**Affiliations:** 1 Songjiang Research Institute, Songjiang Hospital & MOE-Shanghai Key Laboratory for Children’s Environmental Health, Shanghai Jiao Tong University School of Medicine, Shanghai, 201699 China; 2 Institute of Neuroscience, State Key Laboratory of Neuroscience, Center for Excellence in Brain Science and Intelligence Technology, Chinese Academy of Sciences, Shanghai, 200031 China; 3 MOE-Shanghai Key Laboratory for Children’s Environmental Health, Xinhua Hospital, Shanghai Jiao Tong University School of Medicine, Shanghai, 200092 China; 4 Clinical Neuroscience Center, Department of Neurology, Ruijin Hospital, Shanghai Jiao Tong University School of Medicine, Shanghai, 200025 China

**Keywords:** Social behavior, Retrosplenial cortex, Hippocampal CA1

## Abstract

The retrosplenial cortex has been implicated in processing sensory information and spatial learning, with abnormal neural activity reported in association with psychedelics and in mouse and non-human primate models of autism spectrum disorders (ASDs). The direct role of the retrosplenial cortex in regulating social behaviors remains unclear. In this work, we reveal that neural activity in the retrosplenial agranular cortex (RSA), a subregion of the retrosplenial cortex, is initially activated, then quickly suppressed upon social contact. This up-down phase of RSA neurons is crucial for normal social behaviors. Parvalbumin-positive GABAergic neurons in the hippocampal CA1 region were found to send inhibitory projections to the RSA. Blocking these CA1-RSA inhibitory inputs significantly impaired social behavior. Notably, enhancing the CA1-RSA inhibitory input rescued the social behavior defects in an ASD mouse model. This work suggests a neural mechanism for the salience processing of social behavior and identifies a potential target for ASD intervention using neural modulation approaches.

**Supplementary Information:**

The online version contains supplementary material available at 10.1007/s12264-023-01172-0.

## Introduction

Social behaviors are essential for the survival and reproduction of mammals [1]. The execution of social behaviors requires the perception of sensory information, salience processing of social-related information, and further integration in the prefrontal cortex [2]. Abnormal social behaviors linked to neuropsychiatric disorders, such as autism spectrum disorder (ASD), seriously affect individuals’ quality of life [3–5].

Recent findings have shown that ketamine treatment increases neural activity in the retrosplenial cortex (RSC) and decreases social behaviors in mice [6]. Abnormal upregulation of neural activity has been reported in the RSC of *MECP2*-overexpressing mice and increased functional connectivity of RSC and other brain regions in non-human primate models for ASD, compared to wild-type (WT) animals [7, 8]. Our group, along with Li and colleagues, have found significant alterations in excitatory and inhibitory synaptic transmission in the RSC in various genetic mouse models of ASD [9, 10]. These findings collectively suggest that the RSC plays a role in regulating social behavior.

The RSC consists of two subregions, the granular cortex (RSG) and the agranular cortex (RSA), which are anatomically and functionally distinct [11–13]. The RSC receives synaptic inputs from several areas, including the visual cortex, hippocampus, thalamic nuclei, and the anteroventral nucleus [14–16]. In particular, synaptic inputs to the RSC from the hippocampus are crucial for memory formation and consolidation [17–19]. Intriguingly, our findings indicate that the activity of hippocampal CA1 neurons is causally correlated to social behaviors in MECP2-overexpressing mice, a model of ASD [20].

Salience processing of social *versus* non-social inputs from sensory systems is critical for interactive behaviors with conspecific partners during social-related inter-brain neural activity in the prefrontal cortex [1, 2]. Consequently, we hypothesized that the neural circuit from the hippocampus to the RSC plays a pivotal role in modulating social behaviors through the salience processing of sensory inputs, which may be disrupted by ASD-related genetic mutations.

In this study, we discovered that RSA neuron activity, initially activated but quickly suppressed during social contact, is crucial for social behaviors. Parvalbumin-positive interneurons in the hippocampal CA1 region inhibit the RSA, impacting social behavior. Enhancing this CA1-RSA inhibition improved social interaction in an ASD mouse model, indicating that CA1 to RSA inhibitory input regulates social behavior, possibly by filtering non-social sensory information in the RSA. We not only propose a neural mechanism for the salience processing of social information but also highlight a candidate brain region for ASD intervention using neural modulation approaches.

## Methods and Materials

### Animals

Mice were housed in a temperature-controlled environment (22–24 °C) with *ad libitum* access to food and water. Mice were reared in normal lighting conditions (12-h light/dark cycle). Male and female mice (5–6 weeks old at the time of initial surgery) from the following lines were used: C57BL/6J (The Jackson Lab, Cat# 000664), PV-Cre (B6.129P2-*Pvalb*^*tm1(cre)Arbr*^/J, Cat #:017320), and Vgat-cre (B6J.129S6(FVB)-*Slc32a1*^*tm2(cre)Lowl*^/MwarJ, Cat#:028862); *Mef2c-het* mice were acquired from Qi Zhang’s lab at ZheJiang University. The genotype of Senp1+/− mice was determined by applying two parallel PCRs using the same forward primer from exon 8 of *Mef2c* (5’-ACTTGGCCTCTCTGCTCCACTTG-3’) with different reverse primers: one primed in intron 8 of the *Mef2c* gene (5’-TGTATGCTGCAAGCGTCTGTCG-3’). PCR was carried out using standard techniques. All experiments were approved by the Animal Care and Use Committee of the Institute of Neuroscience, Chinese Academy of Sciences, Shanghai, China (IACUC No. NA-016-2016).

### C-fos Immunostaining

After ~ 12 h social isolation in the home cage, a stranger mouse was delivered into the cage. In the control group, a novel object was put into the cage instead. During the interaction period, the environment was kept quiet. After ~ 1.5 h, the mice were successively anesthetized by isoflurane, and sacrificed for C-fos immunostaining.

### *In Vivo* Optogenetic Stimulation

After ~ 12 h social isolation in the home cage, a stranger mouse was delivered into the cage. In the continuous-activation experiment, once the stranger mouse was delivered into the home cage, the optogenetic stimulation was given using blue light for activation of ChR2, with 20 Hz pulses, and the pulse width was 5 ms. In the sniffing-start experiment, we turned on the light just at the beginning of the sniffing behavior and once the sniffing was over, we immediately turned off the light by hand.

### Calcium Imaging

Mice were allowed to recover for two weeks after injection of AAV virus expressing GCaMP6s. After ~ 12 h social isolation in the home cage, we put the cage with the mouse into the behavioral chamber for recording. After 5 min habituation, a stranger mouse was delivered into the cage, and the Ca^2+^ recording was started immediately using QAXK-FPS-TC-LED (QAXK). Onset points of the sniffing behavior were determined by analyzing the video frame-by-frame and retrieving analog signals with the MatLab program. Photometry data were subjected to minimal processing consisting of only autofluorescence background subtraction. The values of the change in Ca^2+^ transients (ΔF/F) from − 4 s to 10 s (0 s represents the onset of actively touching the body during sniffing) were derived by calculating (F-F0)/F0 for each trial, where F0 was defined as the baseline signals from − 4 s to 0 s with subtraction of the autofluorescence background. After recording, all animals were perfused to confirm the virus expression regions and the optic fiber recording sites.

### *In Vivo* Stereotaxic Injections

Standard stereotactic procedures were applied to mice under anesthesia (Pentobarbital, Sigma, Cat#P3761, 50 mg/kg). Virus and CTB were injected in a volume of 200–400 nL/site at a rate of 20 nL/min using a micro-injector and micro-infusion pump (PHD 2000, Harvard Apparatus) into the RSA according to standard mouse brain atlas (Paxinos and Franklin Mouse Brain Atlas, 2nd edition) at the following coordinates: anteroposterior (AP), − 1.82 mm; mediolateral (ML), 0.5 mm; dorsoventral (DV), − 0.45 mm; and into CA1 at the following coordinates: AP, − 1.80 mm; ML, 1.30 mm; DV, − 1.5 mm. Ten minutes after the viral injection, the glass pipette was slowly withdrawn to avoid the back-flow of the virus. RetroAAV virus was injected in a volume of 20 nL/site at a rate of 5 nL/min. Mice were allowed 3–4 weeks for viral expression before the behavioral tests. The viruses and titers used in this study were as follows:

AAV2/9-CaMKII-GCaMP6s-WPRE-pA (1.21E + 12 v.g./mL), AAV2/9-hSyn-DIO-GCaMP6s-WPRE-pA (1.36E + 12 v.g./mL), AAV2/9-CaMKII-ChR2-EYFP-WPRE-pA (1.54E + 12 v.g./mL), AAV2/9-hSyn-GtACR-mCherry-WPRE-pA (1.10E + 12 v.g./mL), AAV2/9-DIO-ChR2-EYFP-WPRE-pA (1.13E + 12 v.g./mL), AAV2/2RetroPlus-hSyn-Cre-mCherry-WPRE-pA (1.52E + 13 v.g./mL), AAV2/9-DIO-mCherry-WPRE-pA (1.15E + 12 v.g./mL), AAV2/9-DIO-hM4D-mCherry-WPRE-pA (1.35E + 12 v.g./mL), AAV2/9-PV.Promoter.S5E2-hChR2(H134R)-mCherry-WPRE-pA (3.2E + 11 v.g./mL), and AAV2/9-PV.Promoter.E29E2-mCherry-WPRE-pA (3.1E + 11 v.g./mL).

### Slice Electrophysiology

Mice were anesthetized with sodium pentobarbital (Sigma, Cat#P3761, 50 mg/kg) 4 weeks after surgery. Coronal brain slices were cut at 300 μm on a vibratome (VT1200S, Leica) in an ice-cold artificial cerebrospinal fluid (aCSF) (in mmol/L, 125 NaCl, 3 KCl, 2 CaCl_2_, 2 MgSO_4_, 1.25 NaH_2_PO_4_, 1.3 NaH_2_PO_4_, 1.3 Na-pyruvate, 26 NaHCO_3_, and 11 glucose, at pH 7.4, 290-310 mOsm) saturated with 95% O_2_ and 5% CO_2_. After ~ 1 h incubation, a slice was transferred into the recording chamber which was constantly perfused with aCSF, and the temperature was controlled at ~ 30 °C by a temperature controller (Warner Instrument Co., USA). Whole-cell recordings were made randomly from neurons in layer IV/V of the RSA; the neurons were visualized under an infrared microscope (Andor) equipped with epifluorescence and infrared-differential interference contrast illumination. Patch pipettes were pulled from borosilicate glass (3–5 MΩ) and filled with a solution consisting of (in mM), 130 K-gluconate, 20 KCl, 10 HEPES, 0.2 EGTA, 4 Mg_2_ATP, 0.3 Na_2_GTP, and 10 Na_2_-phosphocreatine, at pH 7.3 (290–310 mOsm). Series resistance in whole-cell patch-clamp recording was < 30 MΩ. Data were acquired with pClamp9.2 (Molecular Devices) using an AxonMultiClamp 700A amplifier (Molecular Devices), filtered at 2 kHz (low pass), and digitized at 20–100 kHz (Digidata 1322A; Molecular Devices). Before recording, the junction potential was corrected. The data were analyzed with Clampfit 10.3. All chemicals were from Sigma. During electrophysiological recording, a pulse of 5 ms blue or yellow light from a light-emitting diode (LED) source was applied to the acute slice through an Olympus 60× water-immersion lens. The responses evoked by blue light were averaged from 6 sweeps of recording with a 5-s inter-sweep-interval. Recordings were made in voltage-clamp mode, with the command potential set to − 70 mV to record EPSCs. It was then changed to 0 mV to record IPSCs from the same neuron. During recording in CA1, the EYFP-positive neurons were identified by the LED system and whole-cell recordings were made under the current-clamp mode, and a continuous blue light was given to evoke spikes. SR95531 was used at 10 μmol/L.

### *In Vivo* Pharmacogenetic Inhibition

For inhibition of the CA1 PV axons in the RSA, AAV2/9-DIO-hM4D-mCherry-WPRE-pA or AAV2/9-DIO- mCherry-WPRE-pA was unilaterally injected into CA1, and a micro tube (Intracranial Cannula System) was implanted into the superficial layer of the RSA. After 4 weeks of recovery and AAV expression, before the Ca^2+^ imaging experiments, the mice were anesthetized by isoflurane, and 200 nL CNO (1 mmol/L) was infused into the tube at 50 nL/min using a micro-injector and micro-infusion pump (PHD 2000, Harvard Apparatus). Five minutes after the infusion and recovery from anesthesia, the mice were used in the Ca^2+^ imaging and three-chamber experiments.

### Histology and Immunostaining

Mice were anesthetized with sodium pentobarbital (Sigma, Cat#P3761, 50 mg/kg) and perfused with PBS followed by 4% PFA. After perfusion, the brains were post-fixed overnight in 4% PFA at 4 °C and sequentially dehydrated in 30% sucrose/PBS. Then, the brains were embedded in Optimum Cutting Temperature formulation (Sakura, Cat#4583) and cut at 40 µm on a Microtome Cryostat (Leica, CM1950) at − 25 °C. Floating brain sections (40 µm) were rinsed in PBS and then blocked overnight at 4 °C in PBS containing 5% bovine serum albumin (BSA) and 0.2% Triton X-100, followed by incubation with rabbit anti-PV primary antibodies (Abcam, Cat#ab181086; 1:500) at 4 °C overnight and donkey rabbit Alexa Fluor 555 secondary antibodies (Thermo Fisher Scientific, Cat#A-31570, 1:1000) at 4 °C for 2 h. All primary and secondary antibodies were diluted with PBS containing 5% BSA and 0.4% Triton X-100. All brain sections were finally counter-stained with DAPI (Sigma, Cat#d9542, 5 mg/mL, 1:1000). The sections were washed 3×10 min in PBS before incubation with secondary antibodies. For other antibody combinations, sections were rinsed with PBS, blocked, and treated with primary and secondary antibodies as described above (see also KEY RESOURCES TABLE-"Appendix"). Images were captured under a fluorescence microscope (Olympus, VS120, 10×).

### Social Interaction Tests

All the mice used in the behavioral tests were male and handled for > 3 days prior to behavioral tasks. The videos were recorded and analyzed by Noldus, EthoVisionXT 11.5. Before the social approach test, mice were put into the middle chamber for 10 min to habituate. In the home-cage test, a male mouse was left in a cage for ~ 12 h of social isolation, then a stranger mouse was delivered into the cage. During the interaction period, the environment was kept quiet. The social interaction was recorded by a camera. Then the video was analyzed manually. In the social approach test of the three-chamber test, a novel C57BL/6N male mouse (stranger) was put into the left chamber and left for 10 min. In the social novelty test of the three-chamber test, a novel C57BL/6N male mouse was delivered into the right chamber and the video recording was sustained for 10 min. A low light intensity (30 Lux) was used. Then the data were recorded and analyzed by Noldus, EthoVisionXT 11.5.

### Open Field

The mice were handled for 4 consecutive days, 4 min each time to familiarize them with the smell of the experimenter before the test. During the experiment, each mouse was put into the open field (40 × 40 cm^2^) and left for 10 min. The movement of mice was recorded and analyzed by Noldus, EthoVisionXT 11.5.

## Results

### Up-Down Phase of RSA Neuronal Activity During Social Interaction

**Fig. 1:**
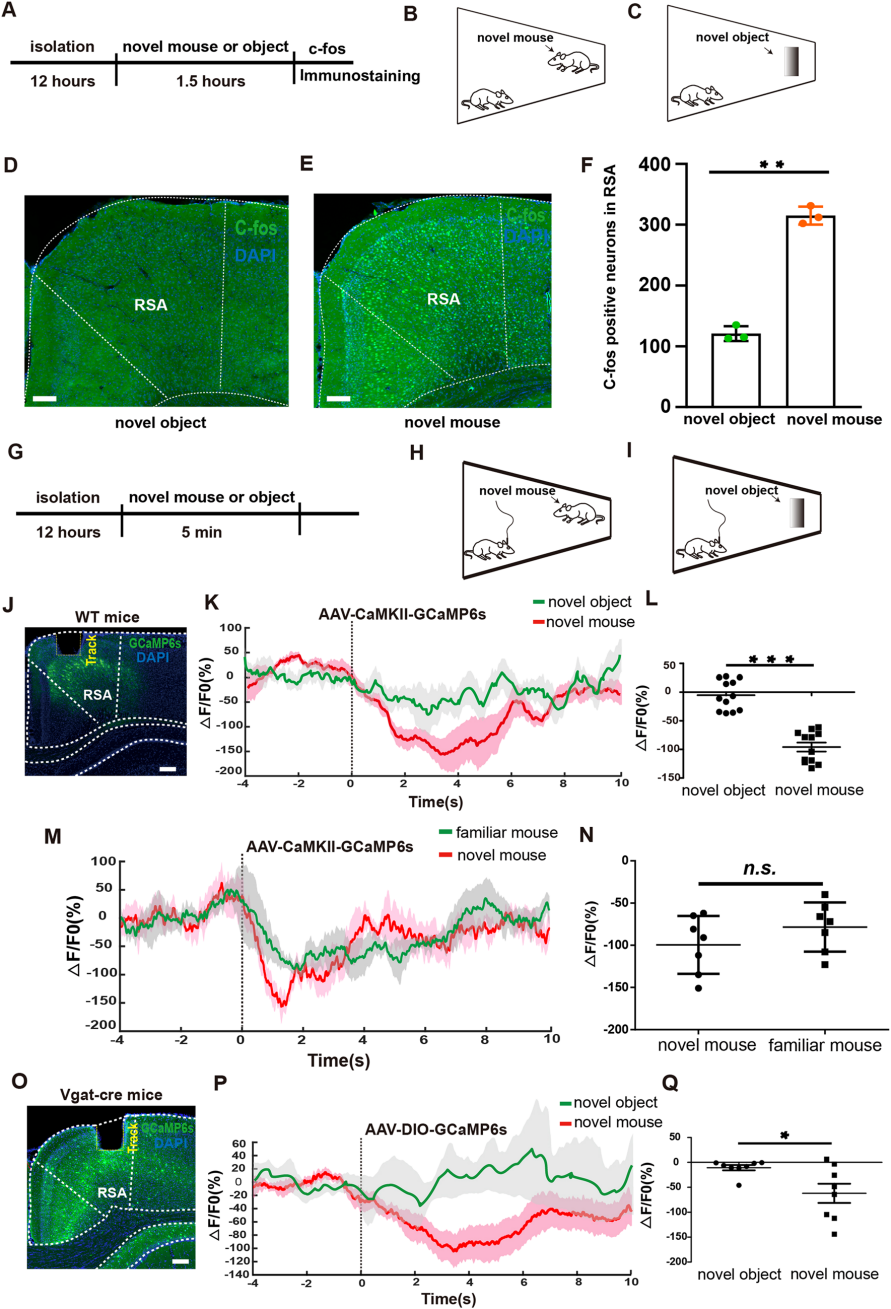
The RSA is activated by social contact and inhibited after sniffing initiation. **A**–**C** Schematic of the home-cage test. **D**, **E** Representative images showing c-fos expression in RSA after interaction with an object (**D**) and a novel mouse (**E**). Scale bars, 300 μm. **F** Numbers of c-fos-positive neurons in the RSA (from **D**, **E**). **G**–**I** Schematic of the home-cage test for the fiber-photometry experiment. **J** Implantation of the optic fiber for fiber photometry in RSA layer IV-V of a WT mouse injected with AAV-CaMKII-GCaMP6s. Scale bar, 250 μm. **K** Mean Ca^2+^ transient associated with social interaction. Solid lines, the mean; shaded areas, SEM (green: object, red: novel); dashed line at 0 s, the time point when mice actively touched the novel mice with the nose. **L** Average △F/F value at 0–6 s from mice with either objects or novel mice (baseline, the mean value at − 4 s-0 s; *n* = 12 mice per group). **M** Mean Ca^2+^ transient associated with the social interaction process. Solid lines, the mean; shaded areas, SEM (green: familiar mouse, red: novel mouse); dashed line at 0 s, the time point when the mice actively touched the novel mice with the nose. **N** Average △F/F value at 0–6 s from mice with either familiar or novel mice (baseline, the mean value at − 4 s-0 s; *n* = 7 mice per group). **O** Implantation of the optic fiber for fiber photometry in the RSA of a Vgat-cre mouse injected with AAV-DIO-GCaMP6s. Scale bar, 250 μm. **P** Mean Ca^2+^ transient associated with the social interaction process. Solid lines, the mean; shaded areas, SEM (green: object, red: novel); dashed line at 0 s, the time point when the mice actively touched the novel mice with the nose. **Q** Average △F/F value at 0–6 s from mice with either objects or novel mice (baseline, the mean value at − 4 s-0 s; *n* = 8 mice per group). *P < 0.05, **P < 0.01, ***P < 0.001.

Initially, we determined whether RSC neurons are activated during social interactions. Immunostaining for c-fos, an immediate-early gene indicative of neural activity, was applied to brain slices from mice that interacted with either novel intruder mice or with objects for 1.5 h in a home-cage experiment (Fig. 1A–C). Mice exposed to novel intruders displayed a significantly increased c-fos level in RSA neurons compared to those exposed to objects, suggesting that RSA neurons are activated upon social interaction (Fig. 1D–F).

The RSC is known to be involved in integrating sensory information [21–23]. To identify which RSC subregion receives input from sensory cortices, we injected retrograde retroAAV-Cre-mCherry virus into the RSA or RSG regions of Ai-9 (Rosa26-CAG-loxp-stop-loxp-tdTomato) mice (Fig. S1A–D). Our findings revealed that the RSA, rather than the RSG, primarily received inputs from the primary visual cortex (V1), suggesting an essential role for the RSA in relaying information to higher centers during social interaction. We further applied anterograde tracing by injecting AAV-hSyn-ChR2-mCherry into V1 (Fig. S1E). A considerable number of ChR2-mCherry-expressing axon terminals were found in the RSA, but not in the RSG (Fig. S1F), consistent with previous findings in rats [13].

Since c-fos protein levels persist for hours after being rapidly induced by neural activity, we aimed to investigate the dynamics of RSA neuronal activity during social interactions. We injected AAV-CaMKII-GCaMP6s virus into the RSA and implanted an optic recording fiber. With this setup, we recorded the real-time Ca^2+^ dynamics in RSA neurons using fiber photometry in layers IV-V of the RSA in WT mice during home-cage interactions (Fig. 1J). Interestingly, the Ca^2+^ signal decreased immediately following a novel mouse sniff, compared to sniffing a novel object (Fig. 1K, L). Moreover, Ca^2+^ signals in the RSA rapidly declined after social contact, regardless of whether the mouse encountered a familiar or a novel conspecific (Fig. 1M, N), indicating that the decrease in neural activity in the RSA is specific to social interaction in mice.

We then investigated whether local GABAergic interneurons inhibit RSA neurons. To test this possibility, we injected the AAV-DIO-GCaMP6s virus into VGAT-ires-Cre mice, specifically labeling GABAergic neurons in the RSA (Fig. 1O). Notably, we recorded a significant decline in Ca^2+^ transients in RSA GABAergic neurons within seconds after social contact (Fig. 1P, Q). Our results demonstrated that both excitatory and inhibitory neurons in the RSA are quickly suppressed by a strong inhibitory input during sniffing, despite RSA neurons being initially activated upon social contact.

### Inhibition of RSA Neurons After Social Contact Promotes Social Behavior

**Fig. 2:**
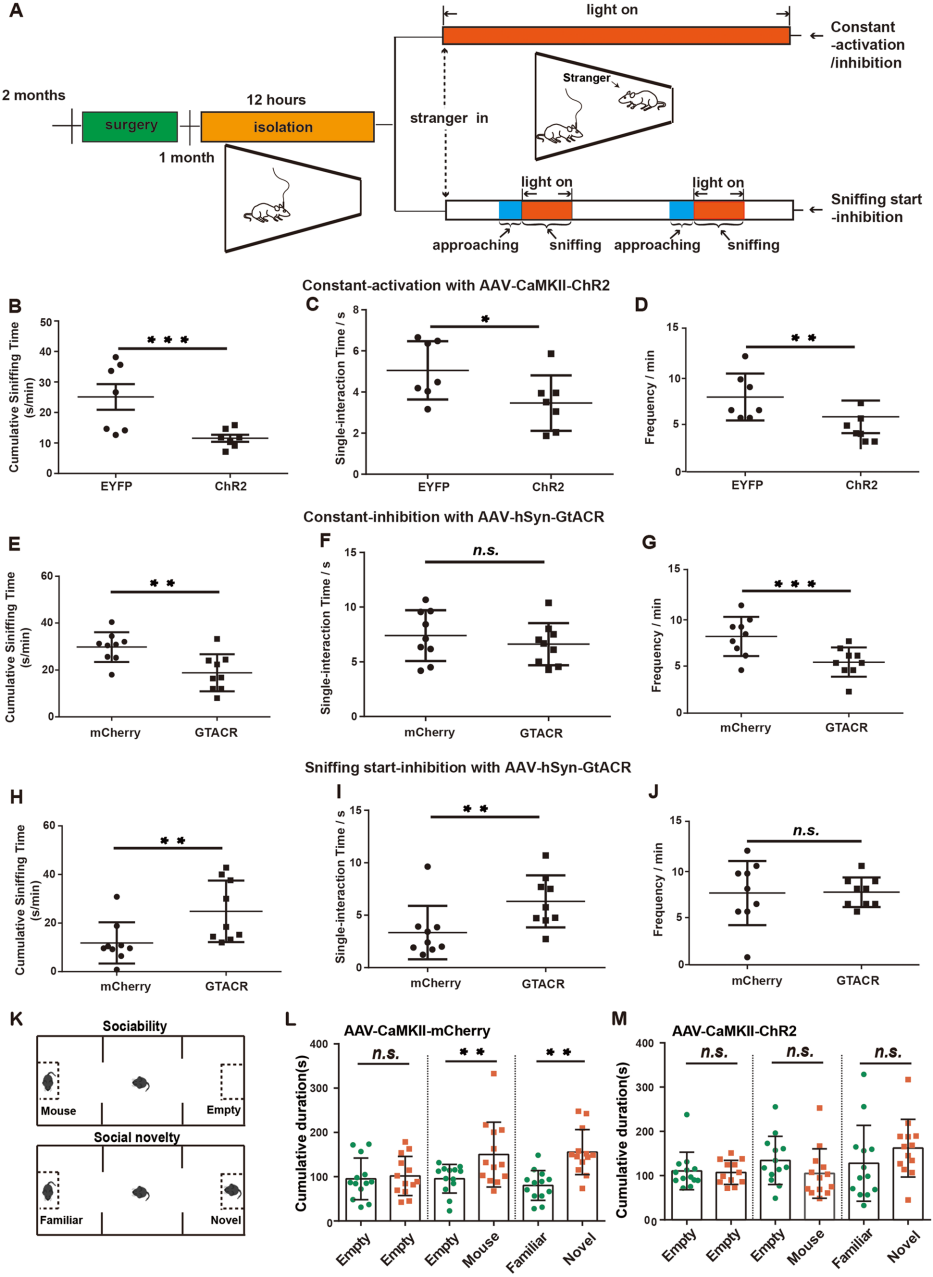
Sniffing start-inhibition of the RSA facilitates home-cage social behavior. **A** Schematic of the optogenetic manipulation in the RSA of mice injected with AAV virus during social interaction in home-cage tests. **B**–**D** Cumulative sniffing time (**B**), single-interaction time (**C**), and sniffing frequency (**D**) induced by constant 473 nm light activation in the ChR2 group and the EYFP group (*n* = 7 mice per group). **E**–**G** Cumulative sniffing time (**E**), single-interaction time (**F**), and sniffing frequency (**G**) induced by constant 550 nm light inhibition in the GtACR group and the mCherry group (*n* = 9 mice per group). **H**–**J** Cumulative sniffing time (**H**), single-interaction time (**I**), and sniffing frequency (**J**) induced by sniffing-start 550 nm light inhibition in the GtACR group and the mCherry group (*n* = 9 mice per each group). **K** Schematic of the three-chamber test, including sociability and social novelty tests. **L**, **M** Cumulative duration during social approach and social novelty sessions in the three-chamber test for the mCherry group (**L**) and the ChR2 group (**M**) (*n* = 13 mice per group). *P < 0.05, **P < 0.01, ***P < 0.001. Error bars represent the mean ± SEM. Optogenetic stimulation parameters: 20 Hz, pulse width 5 ms.

To further investigate whether the up-down phase of RSA neurons is essential for social interactive behaviors, we applied optogenetics with fine temporal resolution to manipulate neural activity. We unilaterally injected AAV-expressing channelrhodopsin2 (ChR2)-EYFP or *Guillardia* theta anion channel rhodopsin (GtACR)-mCherry into the RSA for precise temporal control of RSA neuronal activity (Fig. 2A). After 12 h of isolation, we examined how manipulating neural activity in RSA affected social interactions between a home-cage and a novel mouse. When we constantly activated excitatory RSA neurons during social interaction by stimulating ChR2, mice displayed significantly decreased social interaction, as measured by cumulative sniffing time, interaction time, and interaction frequency, compared to AAV-EYFP-expressing mice (Fig. 2B–D).

We then explored whether inhibiting RSA neurons affects mouse social behaviors. Following AAV-hSyn-GtACR-mCherry injection into the RSA region, we optogenetically inhibited RSA neurons by photostimulation at various stages of social interaction (Fig. 2A). First, we induced constant inhibition by applying 550 nm light to RSA neurons. Interestingly, this resulted in significantly reduced cumulative sniffing time and sniffing frequency of mice with novel intruder mice, indicating that constant blockade of RSA neural activity negatively impacts social behaviors (Fig. 2E–G). Next, we mimicked the down phase by inhibiting neural activity immediately after sniffing initiation (sniffing start-inhibition) (Fig. 2A). Remarkably, both cumulative and single interaction times with novel mice increased significantly following sniffing start-inhibition (Fig. 2H–J), suggesting that suppressing RSA neural activity immediately after social contact enhances social interactive behaviors.

In addition, we determined whether constant RSA neuron activation affects mouse social behavior in the classic three-chamber test paradigm (Fig. 2K). Consistent with our previous findings, RSA neuronal activation led to a complete loss of sociability and social novelty preference (Fig. 2L, M). We also found that continuous RSA activation did not alter mouse anxiety levels in the open-field test (Fig. S2A, B). These data demonstrated that constant RSA activation strongly inhibits social interaction behaviors in mice.

In conclusion, we determined that constant activation or inhibition of RSA neurons during social interaction impairs mouse social behaviors, whereas suppressing RSA neurons immediately after social contact is necessary for proper social interactive behaviors. Abnormalities in sensory information processing have been identified in ASD patients and animal models [24], and correcting genetic defects in the sensory system has been shown to rescue behavioral defects in mouse models of ASD. We hypothesize that the crucial suppression step in the RSA may represent a filtering process that blocks non-social information within sensory inputs during social interaction.

### PV-Positive Neurons in Hippocampal CA1 Project to RSA

**Fig. 3:**
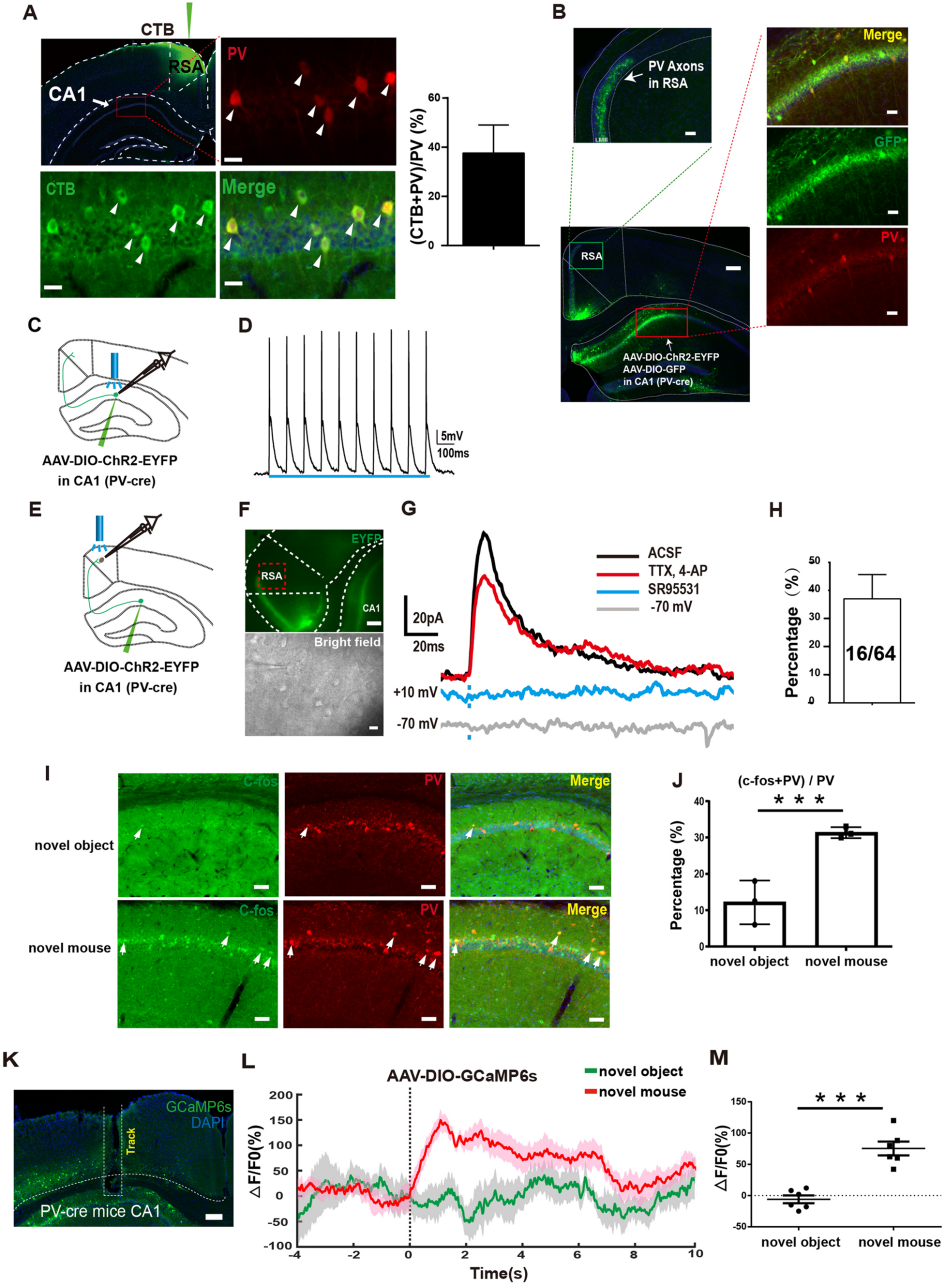
CA1 PV-positive neurons project to the RSA and are activated by social behavior. **A** Left: representative images showing retrograde tracing of RSA neurons with CTB. Right: percentage of CTB and PV double-positive neurons in total PV-positive neurons in CA1. Scale bars, 750 μm (upper left panel), 10 μm (three magnified areas of CA1). **B** Upper left: axon terminals of the CA1 PV+ neurons in the RSA, scale bar, 80 μm. Lower left: the CA1 injection site of the virus; scale bar, 600 μm. Right: magnified regions of CA1, scale bars, 20 μm. **C** Schematic of patch-clamp recording from CA1 PV-positive activated by 473 nm light in PV-cre mice injected with AAV-DIO-ChR2-EYFP into CA1. **D** Spikes recorded from EYFP-labeled neurons in CA1 of PV-cre mice activated by 473 nm light. **E** Schematic of patch-clamp recording from RSA neurons and simultaneous activation of CA1 PV axon terminals using 473 nm light. **F** Representative images of the patch-clamp recording under the infrared microscope illuminated by epifluorescence (upper panel), scale bar, 400 μm, and infrared-differential interference contrast illumination (bright field), scale bar, 10 μm. **G** Representative traces (upper) of IPSCs recorded from RSA neurons activated by 473 nm light with a + 10 mV holding potential which is abolished by the GABA_A_ receptor antagonist SR95531 (green trace), and partially blocked by TTX and 4-AP (red trace). Lower; representative trace recorded from an RSA neuron activated by 473 nm light with a − 70 mV holding potential. **H** Evoked IPSCs were identified on 16 neurons of 64 neurons recorded from 5 mice. **I** Representative images of c-fos expression in CA1 PV+ neurons from mice with objects (upper panels) and novel mice (lower panels) in the home-cage test. Scale bars, 20 μm. **J** Percentages of PV and c-fos double-positive neurons in total PV-positive neurons in mice with new objects or with novel mice (*n* = 18 sections from 3 mice per group). **K** Implantation of an optic fiber for fiber photometry in CA1 of a PV-cre mouse injected with AAV-DIO-GCaMP6s. Scale bar, 250 μm. **L** Mean Ca^2+^ transient associated with the social interaction process. Solid lines, the mean; shaded areas, SEM (green: novel object, red: novel mouse); dashed line, the 0 s time point when the mice actively touched the novel mice with the nose. **M** Average △F/F value at 0-6 s from mice with either a novel object or a novel mouse (baseline is the mean value of − 4 s-0 s (*n* = 6 mice per group). ***P < 0.001.

We next explored the origin of inhibitory inputs to RSA neurons during social interaction. First, we conducted retrograde tracing by injecting the cholera toxin B subunit (CTB) into the RSA and observed numerous labeled neurons in dorsal CA1 (Fig. 3A). We then used immunostaining to identify the subtype of labeled neurons. Intriguingly, we discovered that ~ 38% of the PV-positive neurons in CA1 were labeled by CTB (Fig. 3A), suggesting that PV-positive neurons in CA1 project to the RSA. We further confirmed the synaptic connection between PV-positive neurons in CA1 and the RSA by injecting retroAAV-hSyn-Cre-mCherry into the RSA and AAV-DIO-GFP into CA1 (Fig. S3A, B), revealing that 23% of all the GFP-positive neurons in CA1 were double-positive for PV (Fig. S3C–E).

To label axon terminals and somata, we injected Cre-dependent AAV-DIO-ChR2-EYFP and AAV-DIO-GFP into CA1 of PV-Cre mice (Fig. 3B). ChR2-expressing axons were observed in layers II–III of RSC neurons, indicating that PV-positive neurons send extensive synaptic termini to the RSC (Fig. 3B). We verified this observation by demonstrating that almost all GFP-positive neurons colocalized with PV signals.

To determine whether CA1 PV-positive neurons form functional synaptic connections with RSA neurons, we used an optogenetic-electrophysiological approach to record synaptic currents in brain slice preparations. After injecting Cre-dependent AAV-DIO-ChR2-GFP virus into the CA1 of PV-Cre mice, we made whole-cell recordings from layer IV–V neurons of the RSA (Fig. 3C). Wide-field photostimulation of ChR2-expressing axons at 473 nm revealed that RSA neurons exhibited outward inhibitory postsynaptic currents (IPSCs) at a command voltage of − 10 mV, but no inward excitatory postsynaptic currents when held at − 70 mV (Fig. 3D, E). Moreover, the IPSC was fully blocked by the GABA_A_ receptor antagonist SR95531, indicating mediation by GABA_A_ receptors (Fig. 3E). We recorded IPSCs in 16 out of 64 neurons (5 mice; Fig. 3F). We then made whole-cell recordings from ChR2-EYFP-expressing neurons in CA1 with photostimulation (Figs 3G, S3F) and found the fast-spiking pattern characteristic of the PV-positive GABAergic neurons, confirming the specificity of genetic labeling in mice (Fig. 3H).

We next assessed whether PV-positive neurons in CA1 are responsive to social interaction by applying immunostaining with c-fos in brain slices from mice interacting with either novel objects or novel mice. We found that the c-fos level of PV-positive neurons in CA1 was significantly higher in mice with novel partners than in those with novel objects (Fig. 3I, J).

In addition, we evaluated the responsiveness of CA1 PV-positive neurons to social interaction by injecting the AAV-DIO-GCaMP6s virus into CA1 of PV-cre mice (Fig. 3K). We found that Ca^2+^ signals in CA1 PV-positive neurons significantly increased when mice interacted with novel mice compared to novel objects, indicating that PV-positive neurons in CA1 specifically respond to social interaction (Fig. 3L, M). Thus, we concluded that a significant portion of PV-positive neurons in CA1 project to the RSA and are activated by a social stimulus. We hypothesize that these neurons play a critical role in inhibiting the RSA neurons during social behavior.

The ventral CA1 has been implicated in social memory [25]. In particular, PV-positive neurons in the ventral CA1 have been found to play a pivotal role in social memory [26]. Therefore, we sought to determine whether PV-positive neurons in the ventral CA1 also project to the RSA. Initially, we applied retrograde tracing by injecting CTB into the RSA and found no labeled neurons in the ventral CA1 region (Fig. S3G, H). We then injected AAV-DIO-ChR2-EYFP into the ventral CA1 of PV-cre mice and observed no labeled terminals in the RSA and RSG regions (Fig. S3I, J). We conclude that PV-positive neurons in the ventral CA1 do not project to the RSC [25, 26].

### Inhibitory Input from CA1 PV Neurons to the RSA is Crucial for Social Behavior

**Fig. 4:**
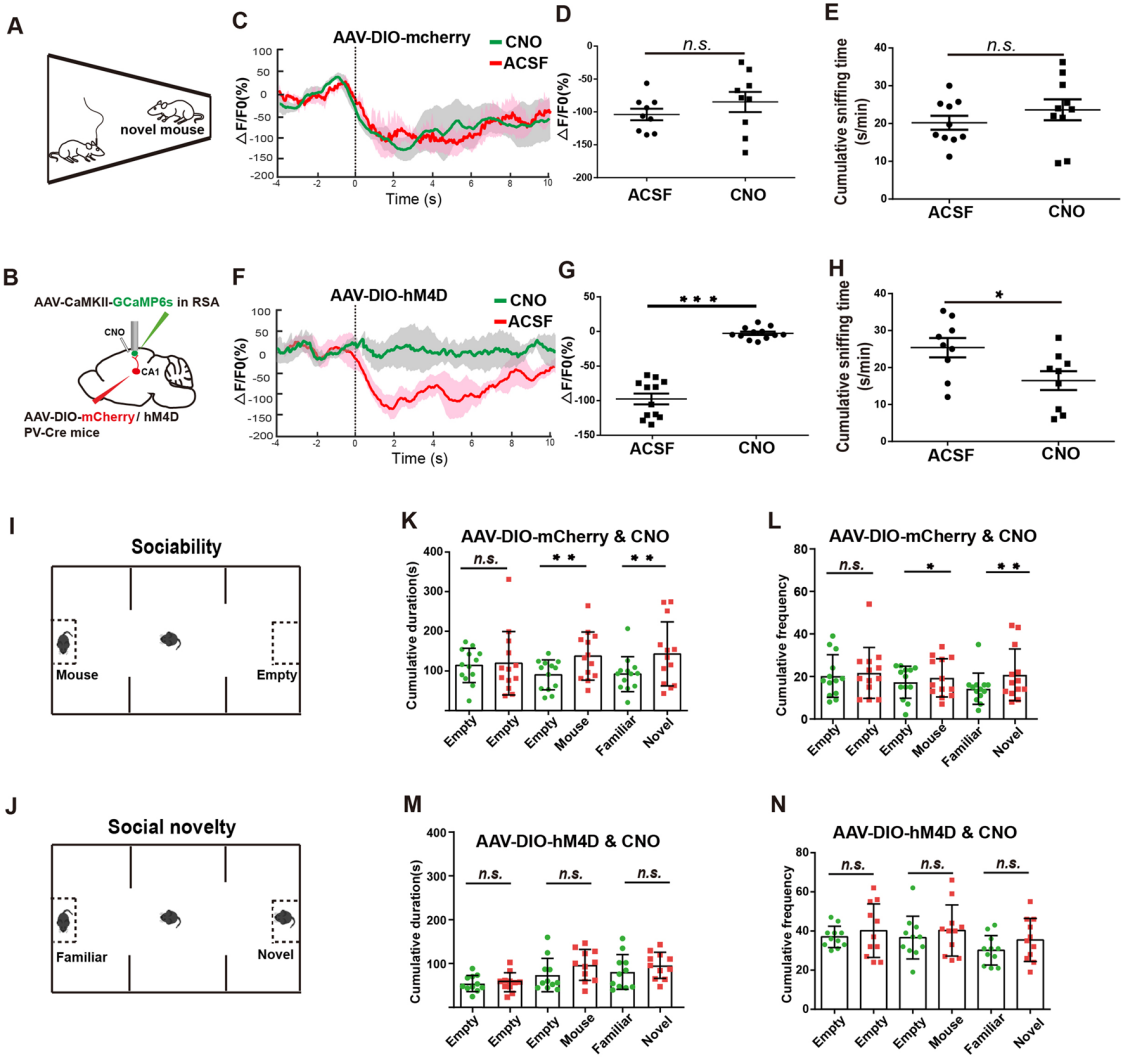
Blockade of the inhibitory projection from PV-positive neurons in CA1 to RSA abolishes the inactivation in RSA during social behavior and leads to a social deficit. **A**, **B** Schematic of the set-up for the home-cage test and the pharmacogenetic manipulation with fiber-photometry recording from RSA excitatory neurons. AAV-DIO-mCherry/hM4D was injected into CA1 of PV-Cre mice and AAV-CaMKII-CCaMP6s was injected into the RSA, with implantation of the optic recording fiber and pipette for CNO infusion. **C** Ca^2+^ transient in mice injected with AAV-DIO-mCherry during social interaction (*n* = 9 mice per group). Solid lines, mean values; shaded areas SEM (green: CNO, red: ACSF); dashed line, the 0 s time point when the experimental mice actively touched the novel mice with the nose. **D** Average △F/F value at 0-6 s (baseline is the mean value at − 4 s-0 s) from mice in the ACSF group and the CNO group (*n* = 9 mice per group). **E** Cumulative sniffing time in the ACSF group and the CNO group in the home-cage test (*n* = 10 mice per group). **F** Ca^2+^ transient of mice injected with AAV-DIO-hM4D during social interaction (*n* = 12 mice per group). Solid lines, the mean; shaded areas, SEM (green: CNO, red: ACSF). **G** Average △F/F value at 0-6 s (baseline is the mean value at − 4 s-0 s) from the ACSF group and the CNO group (*n* = 9 mice per group). **H** Cumulative sniffing time in the ACSF group and the CNO group in home-cage tests (*n* = 9 mice per group). **I**, **J** Schematic of sociability (**I**) and social novelty (**J**) sessions of the three-chamber test. **K**, **L** Social time (**K**) and frequency (**L**) in the three-chamber test for the mCherry group injected with CNO (*n* = 13 mice per group). **M**, **N** Social time (**M**) and frequency (**N**) in the three-chamber test for the hM4D group injected with CNO (*n* = 11 mice per group). *P < 0.05, **P < 0.01, ***P < 0.001.

To investigate the role of the inhibitory input from CA1 PV-positive neurons to the RSA for social behavior, we used a chemogenetic approach by injecting AAV-DIO-mCherry or AAV-DIO-hM4D into CA1 of PV-Cre mice and implanting a cannula into the superficial layer of the RSA for local hM4D activation *via* clozapine-N-oxide (CNO) infusion (Fig. 4A, B). Simultaneously, we injected AAV-CaMKII-GCaMP6s into the RSA and implanted an optical fiber in layer IV–V of the RSA to monitor the neural activity in the RSA (Fig. 4B). We first tested the efficacy of hM4D-mediated inhibition by intraperitoneal CNO injection and observed the complete abolition of the downward phase of the Ca^2+^ signal in the RSA following social contact in the hM4D groups, compared to the mCherry group (Fig. S4A, B).

During the home-cage test, we locally injected CNO into the RSA *via* the implanted cannula and recorded the Ca^2+^ transients of RSA neurons during social interaction (Fig. 4A, B). Consistent with previous findings, the falling phase of the Ca^2+^ transients during social interaction was completely blocked in the hM4D-expressing group, but not in the mCherry-expressing group (Fig. 4C, D, F, G). Surprisingly, social interaction was significantly impaired by blocking the inhibitory input from CA1 PV-positive neurons to the RSA in the hM4D-expressing group, compared to the mCherry-expressing group (Fig. 4E, H).

To further investigate whether the CA1-PV-RSA inhibitory projection influences social behaviors, we applied the classic three-chamber test to measure sociability and social novelty preference (Fig. 4I, J). After CNO treatment, we measured the cumulative duration and frequency of social interactions and found that both sociability and social novelty preference were significantly impaired in the hM4D-expressing group, but not in the control group expressing only mCherry (Fig. 4K–N).

These data indicate that inhibitory input from CA1 PV-positive neurons to the RSA contributes significantly to the suppression of RSA neurons following social contact and is essential for proper social interactive behaviors.

### Activation of the PV^+^ Inhibitory Projection from CA1 to the RSA Rescues the Social Deficit in an ASD Mouse Model

**Fig. 5:**
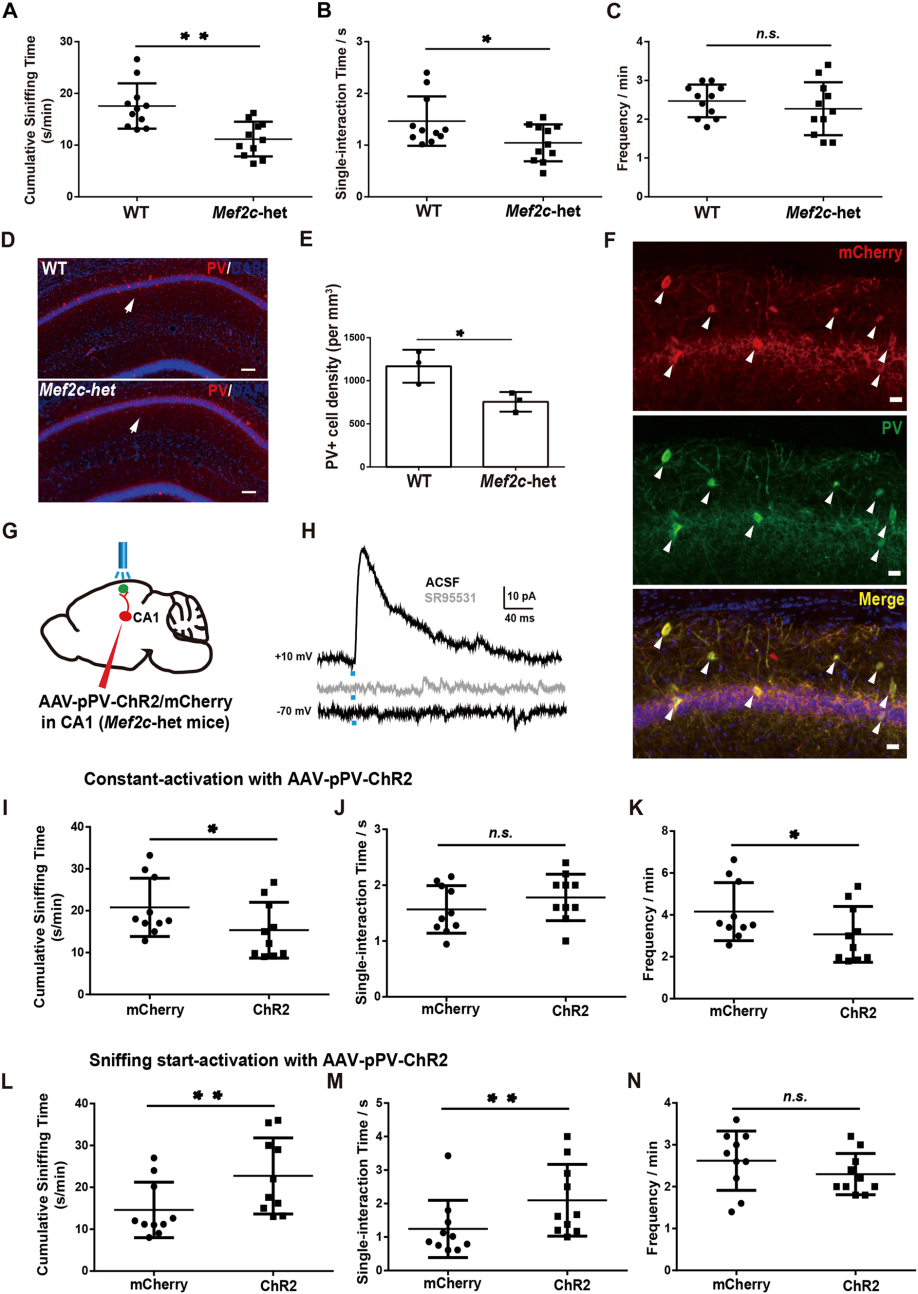
Activating the inhibitory projection from CA1 PV-positive neurons to the RSA after sniffing initiation rescues the social deficit in *Mef2c*^+/−^ mice. **A**–**C** Cumulative sniffing time (**A**), single-interaction time (**B**), and sniffing frequency (**C**) in the *Mef2c*^+/−^ group (*Mef2c*-het) and in wild-type littermates (WT). **D** Representative images showing a decrease of the PV-positive neurons in *Mef2c*-het mice (lower panel); scale bars, 50 μm. **E** Density of PV-positive neurons in CA1 from WT and *Mef2c*-het mice. **F** Immunostaining of PV and mCherry in hippocampal sections from *Mef2c*-het mice injected with AAV-PV-ChR2-mCherry. Arrowheads indicate neurons showing both mCherry and PV positive signals. Scale bars, 10 μm. **G** Schematic of optogenetic manipulation of PV-positive input to the RSA in *Mef2c*-het mice. AAV-pPV-ChR2-mCherry was injected at CA1 of *Mef2c*-het mice with the implantation of an optic stimulation fiber in the RSA. **H** Representative trace (upper) of the IPSCs recorded from an RSA neuron activated by 473 nm light with a + 10 mV holding potential; this is abolished by the GABA_A_ receptor antagonist SR95531(gray trace), and a representative trace recorded from RSA neurons activated by 473 nm light at a − 70 mV holding potential (lower). **I**–**K** Cumulative sniffing time (**I**), single-interaction time (**J**), and sniffing frequency (**K**) induced by constant 473 nm light activation in *Mef2c*-het mice injected with either AAV-pPV-ChR2 (ChR2) or AAV-pPV-mCherry (mCherry). **L**–**N** Cumulative sniffing time (**L**), single-interaction time (**M**), and sniffing frequency (**N**) induced by sniffing-start 473 nm light activation in *Mef2c*-het mice injected with either AAV-pPV-ChR2 (ChR2) or AAV-pPV-mCherry (mCherry). *P < 0.05, **P < 0.01. Error bars represent the mean ± SEM.

We next investigated whether enhancing the inhibitory projection from CA1 PV-positive neurons to the RSA can rescue the social deficits in ASD mouse models. Mutations of *MEF2C* have been identified in individuals with ASD and intellectual disabilities [27]. *Mef2c* haploinsufficient (*Mef2c*^+/−^) mice exhibit ASD-like behaviors, such as defects in social interaction [28]. We investigated whether enhancing the CA1 PV-RSA inhibitory pathway can rescue the social deficit phenotype of *Mef2c*^+/−^ mice. We first confirmed previous findings showing that *Mef2c*^+/−^ mice indeed exhibited social deficits in the home-cage experiment (Fig. 5A–C). In addition, the population of PV-positive neurons was decreased in CA1 of *Mef2c*^+/−^ mice (Fig. 5D, E), as previously reported [28].

To specifically target PV-positive neurons in the hippocampal CA1 region of *Mef2c*^+/−^ mice, we used a PV-specific enhancer to drive the expression of either ChR2 (AAV-pPV-ChR2) or mCherry (PV-mCherry) carried by AAV (Fig. 5F) [29]. Through immunostaining with the excitatory marker CaMKII and the inhibitory marker parvalbumin, we found that the PV-specific enhancer faithfully labeled 85% of PV-positive neurons, and no excitatory neurons were labeled (Fig. S5A–I).

To confirm that ChR2-labeled PV-positive neurons in CA1 of *Mef2c*^+/−^ mice project to the RSA, we made whole-cell recordings from RSA neurons with 473 nm photostimulation (Fig. 5G). We discovered that photo-activation of ChR2-expressing axons evoked robust IPSCs in RSA neurons in brain slices, which were completely blocked by SR95531, an antagonist of the GABA_A_ receptor (Fig. 5H). Among 20 recorded RSA neurons, 6 were responsive (Fig. S6A).

We first determined whether manipulation of CA1-PV-RSA circuits might affect social behaviors in WT mice. We injected AAV-pPV-ChR2 into CA1 of WT mice and photostimulated the axon terminals in the RSA (Fig. S6B). We found that constant activation of the CA1-PV-RSA projection during social interaction led to a decrease in the sniffing time of WT mice with another mouse, consistent with the previous finding (Fig. S6C–E). The sniffing start-activation of the CA1-PV-RSA projection after social contact did not further increase the social interaction time in WT mice (Fig. S6F–H), suggesting that the CA1-PV-RSA projection works properly in WT mice during social interactions.

Since the number of PV-positive neurons in CA1 of *Mef2c*^+/−^ mice was significantly lower than that in WT mice (Fig. 5D, E), we hypothesized that enhancement of the CA1-PV-RSA projection with an optogenetic approach could rescue the social defects of *Mef2c*^+/−^ mice. Finally, we photostimulated the RSA of *Mef2c*^+/−^ mice during the home-cage test. We observed that the overall duration and frequency of social interactions decreased further in the PV-ChR2 group of *Mef2c*^+/−^ mice during constant activation sessions, compared to the mCherry-expressing group (Fig. 5I–K). Remarkably, the social interactive time was significantly rescued in *Mef2c*^+/−^ mice when photostimulation to activate ChR2 was given right after sniffing started (Fig. 5L–N). These results demonstrate that activation of the inhibitory projection of PV-positive neurons from CA1 to RSA during social contact is sufficient to rescue the impairment of social interaction in *Mef2c*^+/−^ mice.

## Discussion

Previously, our work and that of others demonstrated that synaptic transmission in RSC neurons is disrupted in several ASD mouse models, including *Senp1*^+/−^ and *Fmr1*^−/y^ mice [9, 10]. Vesuna et al. showed that abnormally elevated neural activity in the RSC of mice caused by ketamine treatment leads to dissociative behaviors, including defects in social interactions in a dose-dependent manner [6]. This evidence strongly suggests that the RSC plays a critical role in animal social interactions.

In this work, we proposed a model in which the inhibitory projection from PV-positive neurons of CA1 to the RSA serves as a salience processing node to filter out non-social information flowing through the RSA from sensory cortices. Although the processing of social information within the RSA requires further investigation, the data presented here revealed neural mechanisms underlying the salience processing of social interaction behaviors. Since the hippocampus is one of the brain regions receiving extensive sensory inputs besides sensory modules, we further hypothesized that the PV-positive neurons in the hippocampal CA1 region may serve as a salience processing switch by inhibiting non-social sensory information in the RSA upon social interaction, even though all RSC neurons are activated upon social contact.

Social behavior is one of the fundamental interactive behaviors in mammals; it can be described as “interactive behaviors within a community” in non-human mammalian species. Within a community, the valence of social behavior is primarily influenced by social hierarchy and mating possibilities, while the salience of social behavior is largely determined by how the brain processes social *versus* non-social information. The processing of social-related information salience is a crucial step for the progression of social interactive behaviors. Furthermore, the salience of social interaction is compromised in people with ASD, a group of brain disorders with strong genetic predispositions [30].

Our findings suggest that defects in salience processing in the RSA may be a causative factor for abnormal social behaviors in ASD patients. Clinically, defects in sensory perception, including the visual and auditory systems, are widely reported in people with ASD. However, whether a defect in sensory perception is one of the causes or consequences of ASD remains debatable [24]. This work suggests that defects in salience processing in the RSA may be a causative factor leading to autistic-like behaviors, as enhancing this pathway rescues the behavioral defects in mouse models of ASD.

The input and output of RSA circuits are quite intriguing. Although the ventral CA1 has been reported to play a critical role in social memory, we found that the ventral CA1 did not project to the RSA at all, suggesting that PV-positive neurons in the dorsal CA1 may be involved in specific circuits governing social behaviors. Further work involves identifying the upstream of CA1-PV^+^ neurons, as well as the downstream of RSA neurons. The most critical question is to determine how social-related information is processed when the RSA is generally repressed during social interaction. More in-depth single-neuron resolution imaging may provide more insightful answers.

In our study, the activation of both excitatory and inhibitory neurons in the RSA during initial social interactions is particularly intriguing. We hypothesize that this initial activation signal, though brief, is potent enough to induce c-fos expression. However, inhibitory input from CA1 PV neurons may soon block non-social information. To substantiate this hypothesis, one would need to monitor Ca^2+^ activity at single-cell resolution in the RSA while manipulating CA1 PV neurons. We contend that experiments with precise timing control are essential to probe this query further.

The RSC in the human brain is easily accessible with neural modulation approaches, such as transcranial magnetic stimulation. Our finding opened an important venue through which one may test this hypothesis by manipulating neural activity in the RSC in people with ASD and exploring potential intervention methods.

## Electronic supplementary material

Below is the link to the electronic supplementary material.

Supplementary file1 (PDF 1494 KB)

## Data Availability

The datasets used and/or analyzed in the current study are available from the lead contact on reasonable request.
