# Supplementary material for: Gating of Social Behavior by Inhibitory Inputs from Hippocampal CA1 to Retrosplenial Agranular Cortex
Source: Neurosci Bull. 2024 Jan 28;40(11):1635–48. doi: 10.1007/s12264-023-01172-0 (PMC11607374; doi:10.1007/s12264-023-01172-0)
Supplement: Supplementary file 1 — Supplementary file1 (PDF 1494 KB) [file 12264_2023_1172_MOESM1_ESM.pdf]

Supplemental information

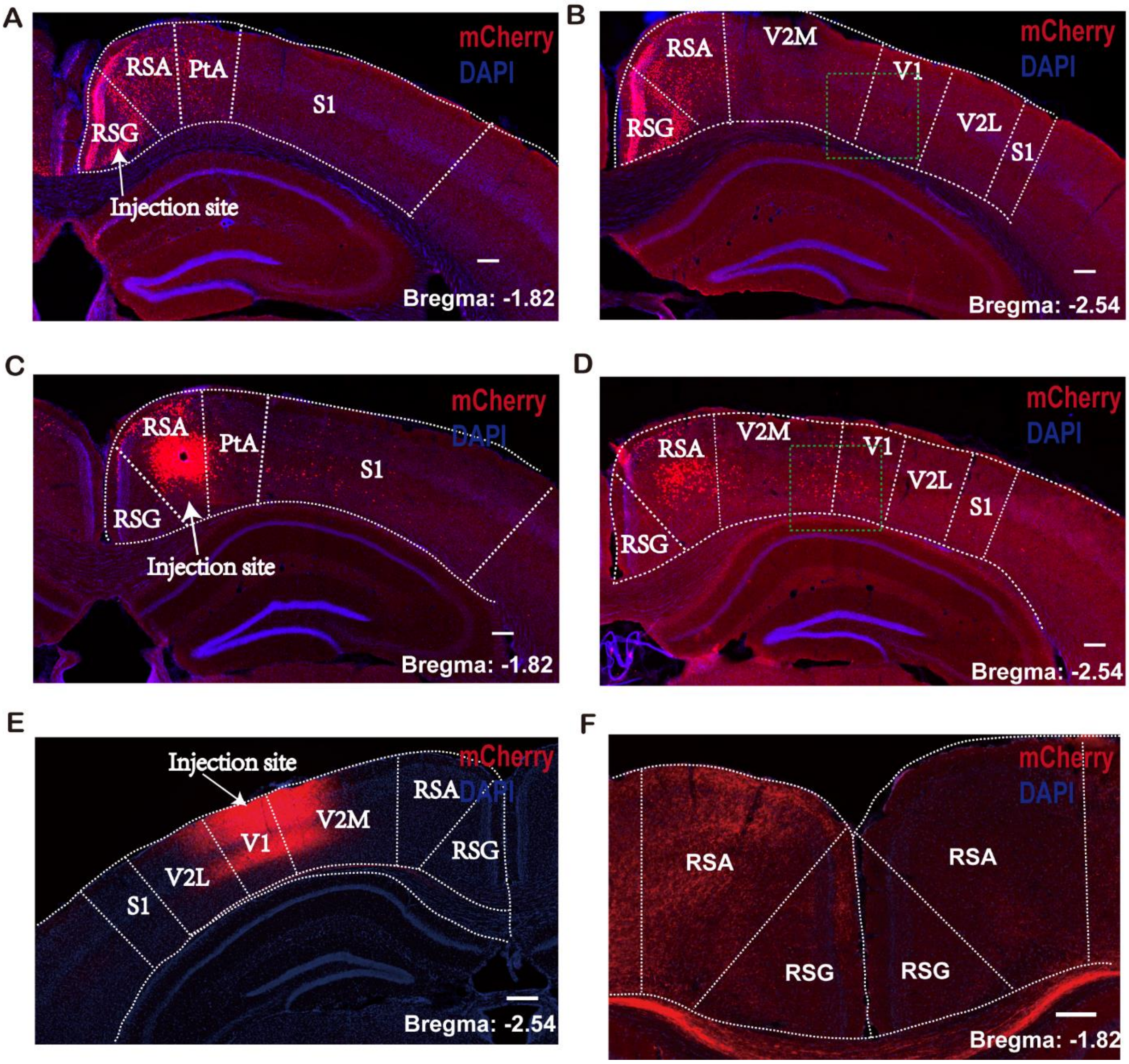

Fig. S1 RSA mainly receives input from primary visual cortex

A-**D** Representative images showing injection sites in the RSG (**A**, **B**) and RSA (**C**, **D**) of retroAAV-cre-mCherry in Ai-9 mice; scale bars, 500 µm. The mCherry-labeled neurons are found in V1 of RSA-injected mice (**D**). **E** Image of the injection site in visual cortex (V1) with AAV-hSyn-ChR2-mCherry; scale bar, 600 µm. **F** ChR2-mCherry labeled axon terminals are mainly found in the RSA; scale bar, 500 µm.

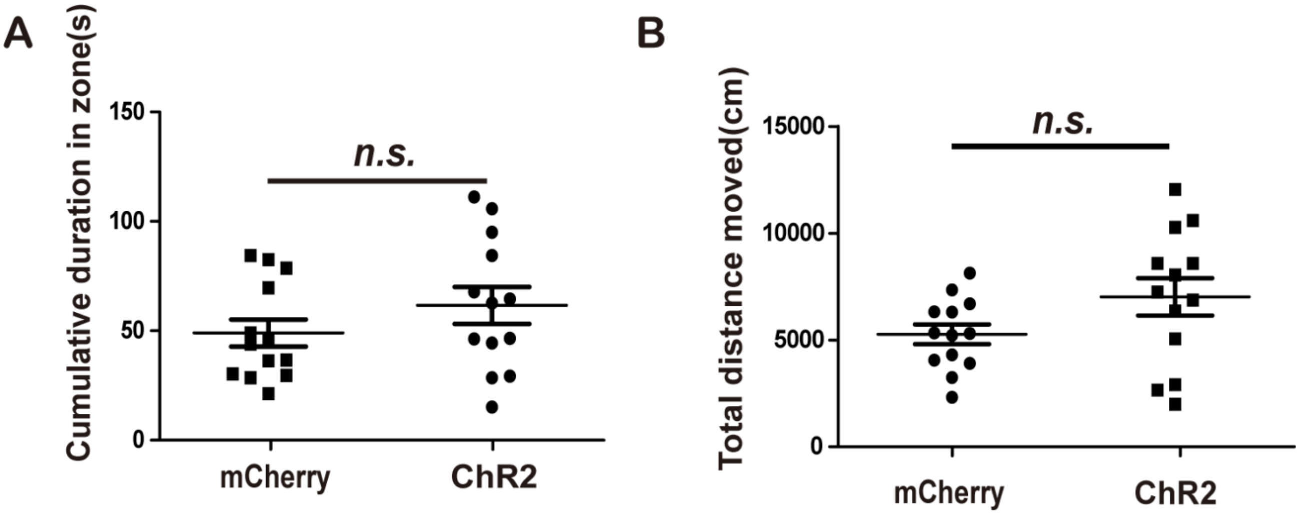

Fig. S2 Activating RSA neurons does not affect the anxiety level of mice. **A**, **B** Cumulative time spent in the central zone (**A**) and total distance moved (**B**) for the mCherry and ChR2 groups in the open-field test (*n* = 13 per group)

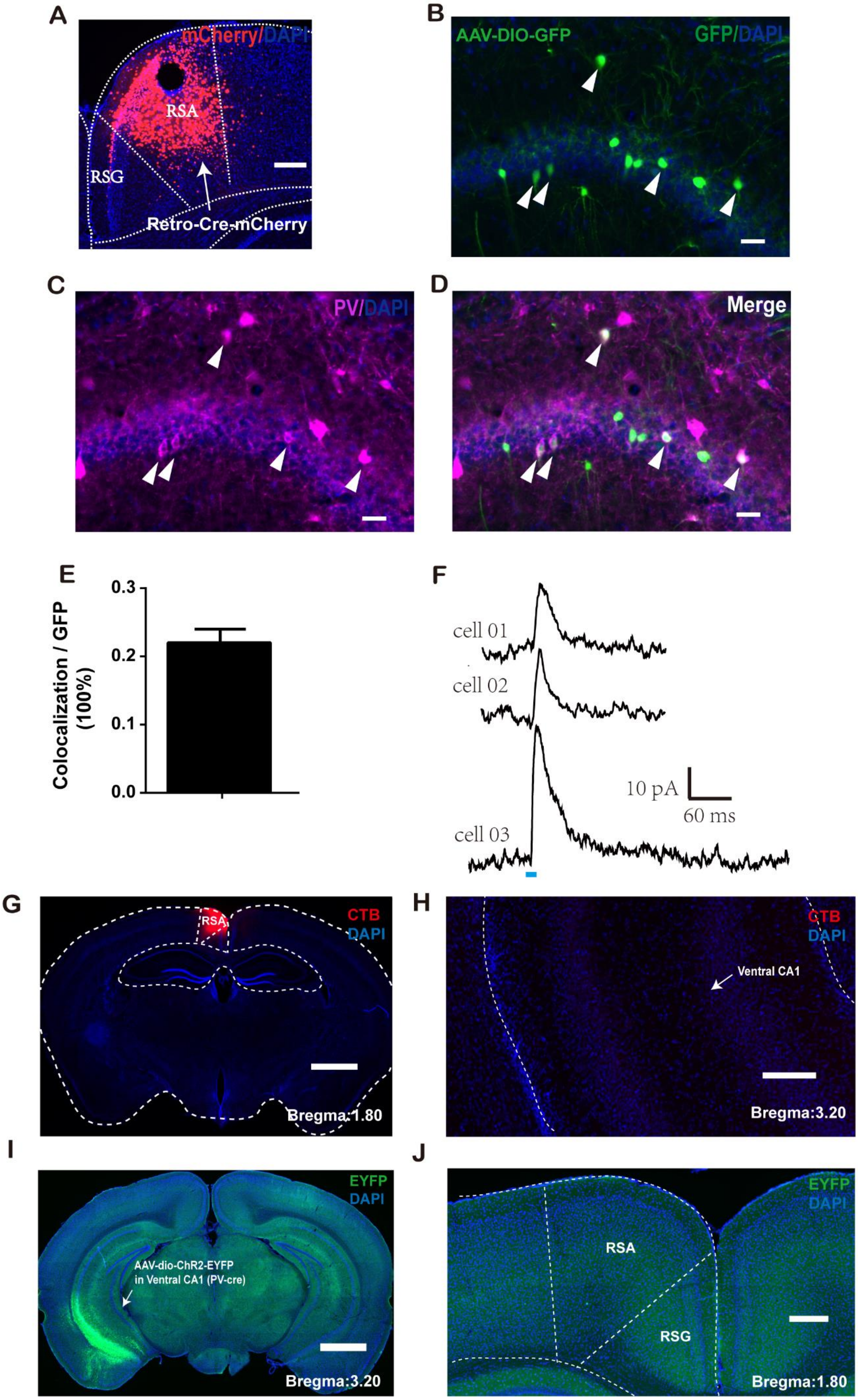
A
mCherry/DAPI
RSA
RSG
Retro-Cre-mCherry
B
AAV-DIO-GFP
GFP/DAPI
C
PV/DAPI
D
Merge
E
Colocalization / GFP (100%)
0.3
0.2
0.1
0.0
F
cell 01
cell 02
cell 03
10 pA
60 ms
G
CTB
DAPI
RSA
Bregma:1.80
H
CTB
DAPI
Ventral CA1
Bregma:3.20
I
EYFP
DAPI
AAV-dio-ChR2-EYFP in Ventral CA1 (PV-cre)
Bregma:3.20
J
EYFP
DAPI
RSA
RSG
Bregma:1.80

Fig. S3 Retrograde labeling of CA1 PV-positive neurons projecting to the RSA.

A Representative image showing the inject site of AAV-Retro-Cre-mCherry in the RSA; scale bar, 500 μm. **B**-**D** The expression of GFP (**B**), PV (**C**), and merged (**D**) in CA1 with injection of AAV-DIO-GFP at the same time; scale bars, 20 μm. Arrowheads indicate the colocalized expression of AAV and the PV staining. **E** Percentage of GFP and PV double-positive neurons in total GFP labeled neurons in CA1. **F** Three typical IPSCs evoked by light. **G** Representative image showing the injection of CTB into the RSA; scale bar, 3 mm. **H** Representative image of the ventral CA1 after CTB injection; scale bar, 1 mm. **I** Representative image showing the injection site of AAV-Dio-ChR2-EYFP in the ventral CA1 of a PV-cre mouse; scale bar, 3 mm. **J** Representative image of the RSA after AAV-Dio-ChR2-EYFP injection; scale bar, 0.8 mm.

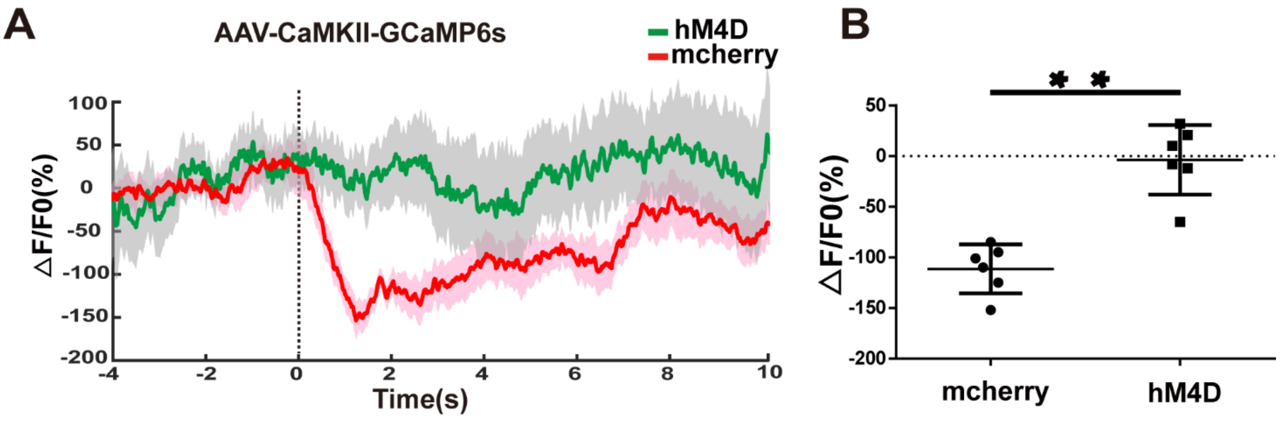

Fig. S4 Blockade of inhibition input by pharmacogenetic manipulations in PV-Cre mice.

A Mean Ca^{2+} transient associated with social interaction. PV-Cre mice with injection of AAV-CaMKII-GCaMP6s into the RSA and either AAV-DIO-hM4D or AAV-DIO-mCherry into CA1. CNO was given through intraperitoneal injection. Solid lines, the mean; shaded areas, SEM (green: hM4D, red: mCherry); dashed line, the 0 s time point when the mice actively touched the novel mice with the nose. **B** Average △F/F value at 0-6 s from hM4D mice and mCherry mice (baseline is the mean value at −4 s-0 s) (n = 6 mice per group). $^{**}P$ <0.01. Error bars represent the mean ± SEM.

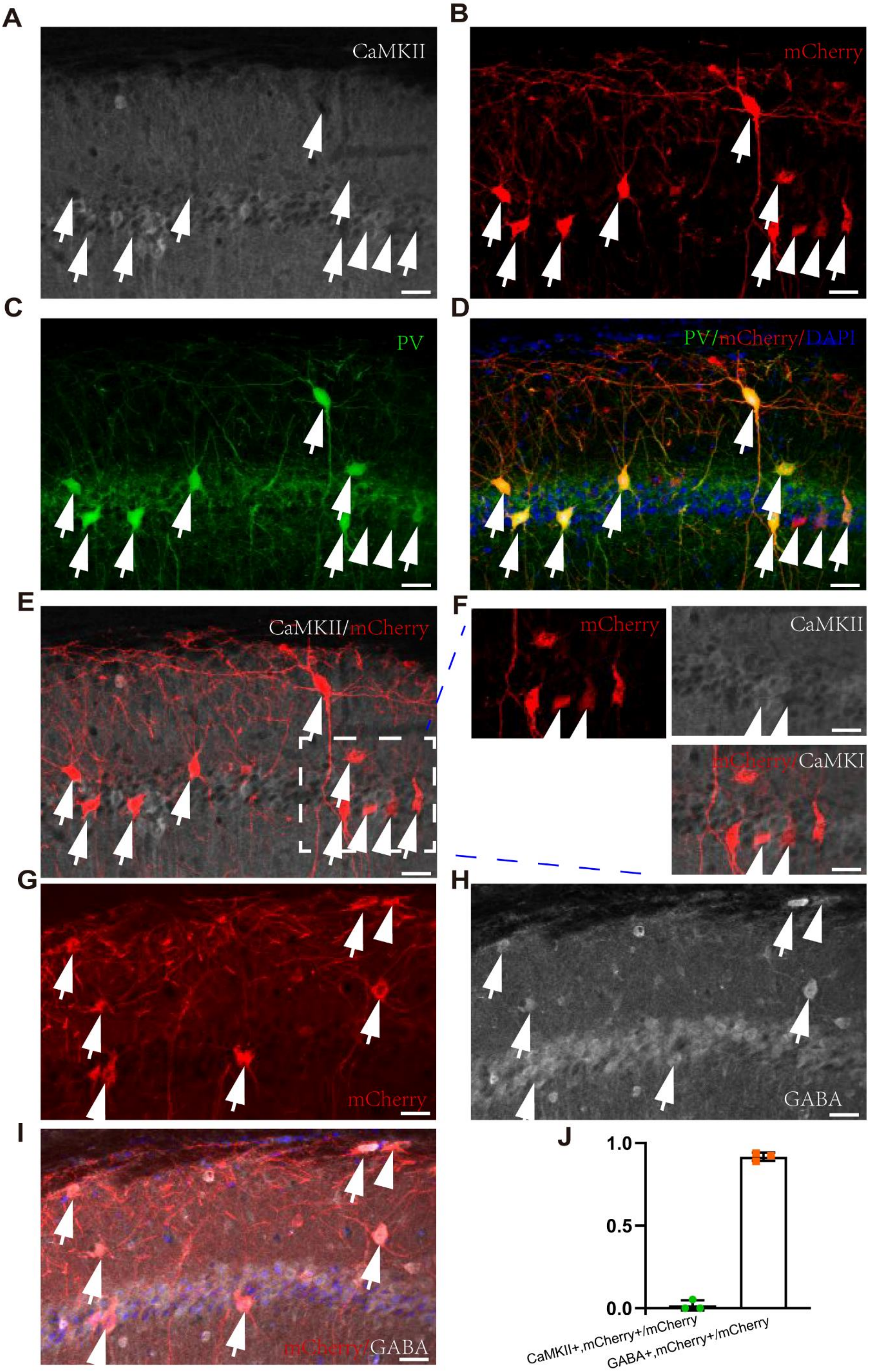
A
CaMKII
B
mCherry
C
PV
D
PV/mCherry/DAPI
E
CaMKII/mCherry
F
mCherry
CaMKII
mCherry/CaMKI
G
mCherry
H
GABA
I
mCherry/GABA
J
1.0
0.5
0.0
CaMKII+,mCherry+/mCherry
GABA+,mCherry+/mCherry

Fig. S5 Immunostaining of CA1 after AAV-pPV-mCherry injection into WT mice.

A-D Representative images of immunostaining in CA1 sections from mice injected with AAV-pPV-mCherry, using antibody against CaMKII (**A**), mCherry (**B**), and PV (**C**), and PV + mCherry merged image (**D**). Arrows indicate mCherry-positive neurons. Arrowheads indicate neurons with mCherry but not PV signals. Scale bars, 35 μm. **E** CaMKII + mCherry merged image. Dashed box is the magnified area. **F** Magnified image of the dashed box in (**E**). Scale bar, 35 μm. **G-I** Representative images of immunostaining in CA1 sections from mice injected with AAV-pPV-mCherry, using antibody against mCherry (**G**), GABA (**H**), and the GABA + mCherry merged image (**I**). Arrows indicate mCherry positive neurons. Scale bars, 35 μm. **J** Percentage of (CaMKII, mCherry)/mCherry, and (GABA, mCherry)/mCherry neurons from (**E** and **I**).

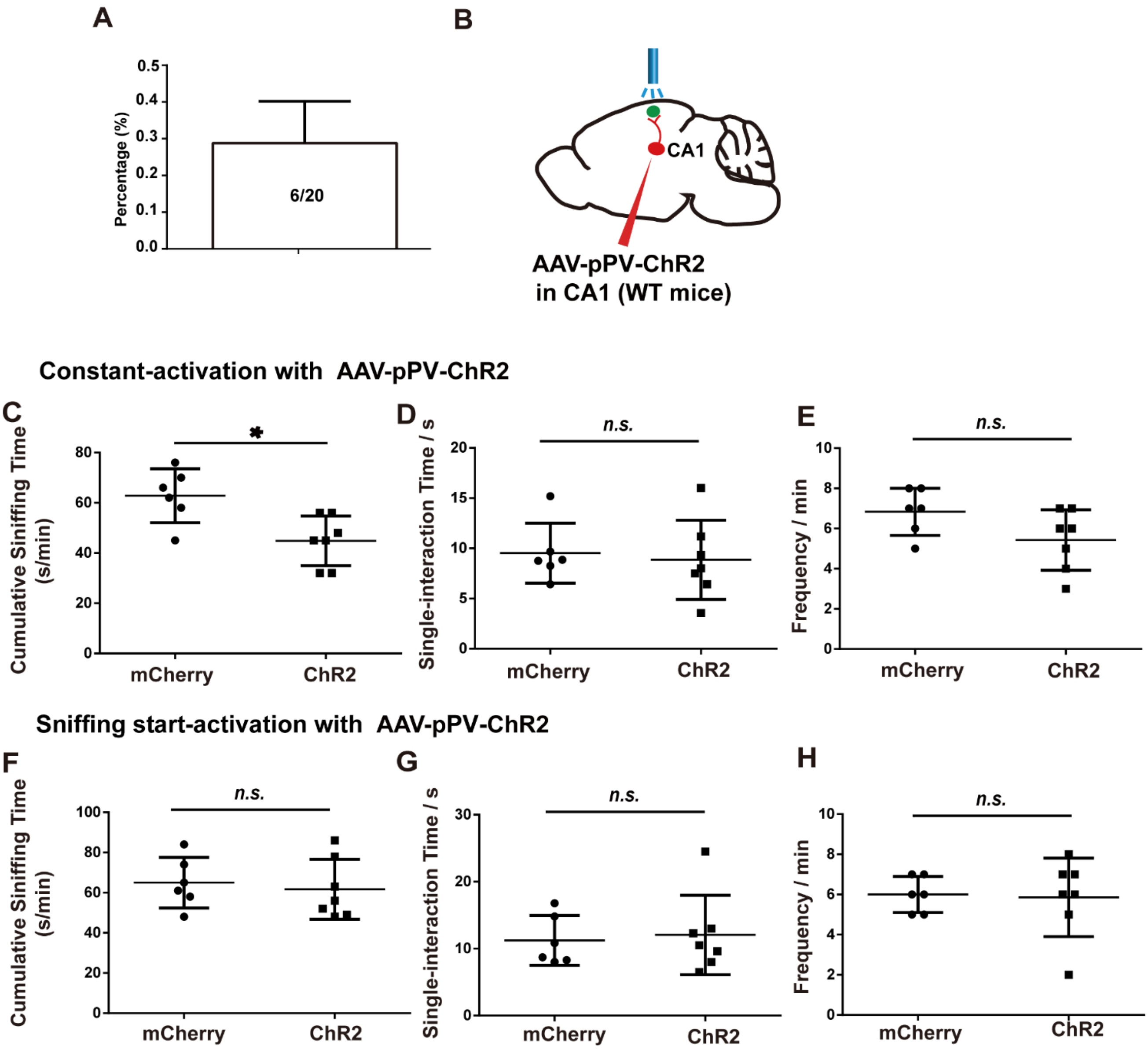

Fig. S6 AAV-pPV-ChR2 targeting the CA1-PV-RSA projection in WT mice.

A Evoked IPSCs were recorded from 6 neurons in a total of 20 neurons from 3 mice. **B** Schematic for optogenetic manipulation of PV-positive input to the RSA in WT mice. AAV-pPV-ChR2-mCherry was injected into CA1 of WT mice with implantation of an optical stimulation fiber in the RSA. **C-E** Cumulative sniffing time (**C**), single-interaction time (**D**), and sniffing frequency (**E**) induced by constant 473 nm light activation in the PV-ChR2 group and the PV-mCherry group. **F-H** Cumulative sniffing time (**F**), single-interaction time (**G**), and sniffing frequency (**H**) induced by sniffing-start 473 nm light activation in the AAV-pPV-ChR2 group and the AAV-pPV-mCherry group. Animal numbers, mCherry, $n = 6$; ChR2, $n = 7$. *P <0.05, Error bars represent the mean ± SEM.
